# Supplementary material for: Endocannabinoid signalling modulates susceptibility to traumatic stress exposure
Source: Nat Commun. 2017 Mar 28;8:14782. doi: 10.1038/ncomms14782 (PMC5379055; doi:10.1038/ncomms14782)
Supplement: Supplementary Information — Supplementary Figures. [file ncomms14782-s1.pdf]

SUPPLEMENTARY FIGURES

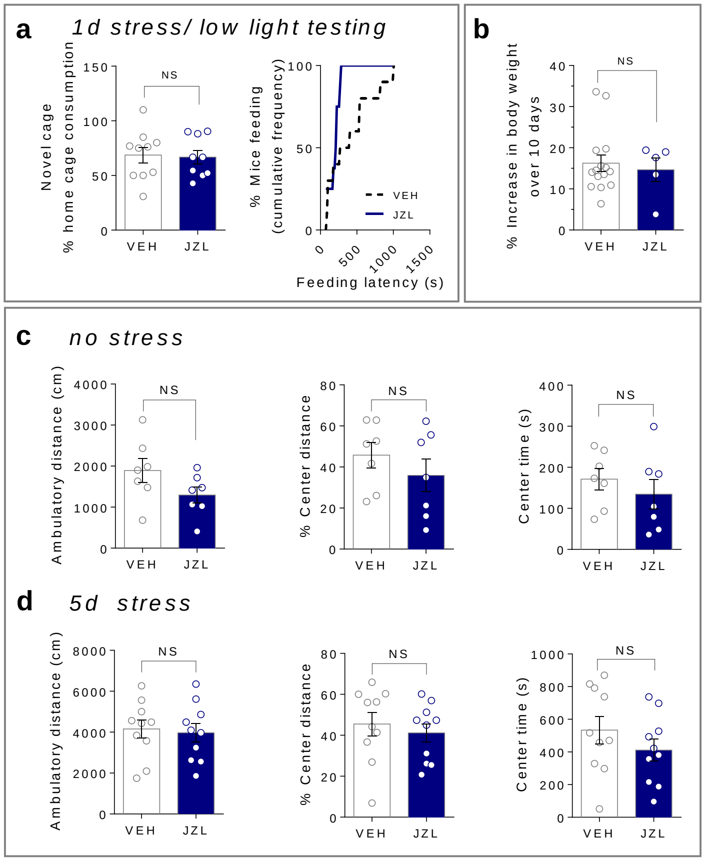

Supplementary Figure 1. 2-AG augmentation does not affect consummatory or exploratory drive. (a) Effect of acute JZL-184 on consumption and latency in a less aversive (low light) NIH novel-cage test 24h after foot-shock stress. **(b)** Effect of 10 days of JZL-184 treatment on body weight in unstressed mice. **(c)** Effects of acute JZL-184 on exploration in a novel open field under control conditions and **(d)** after 5 days of stress. P-values for unpaired two-tailed t-test reported in each panel. Data are presented as mean ± SEM.

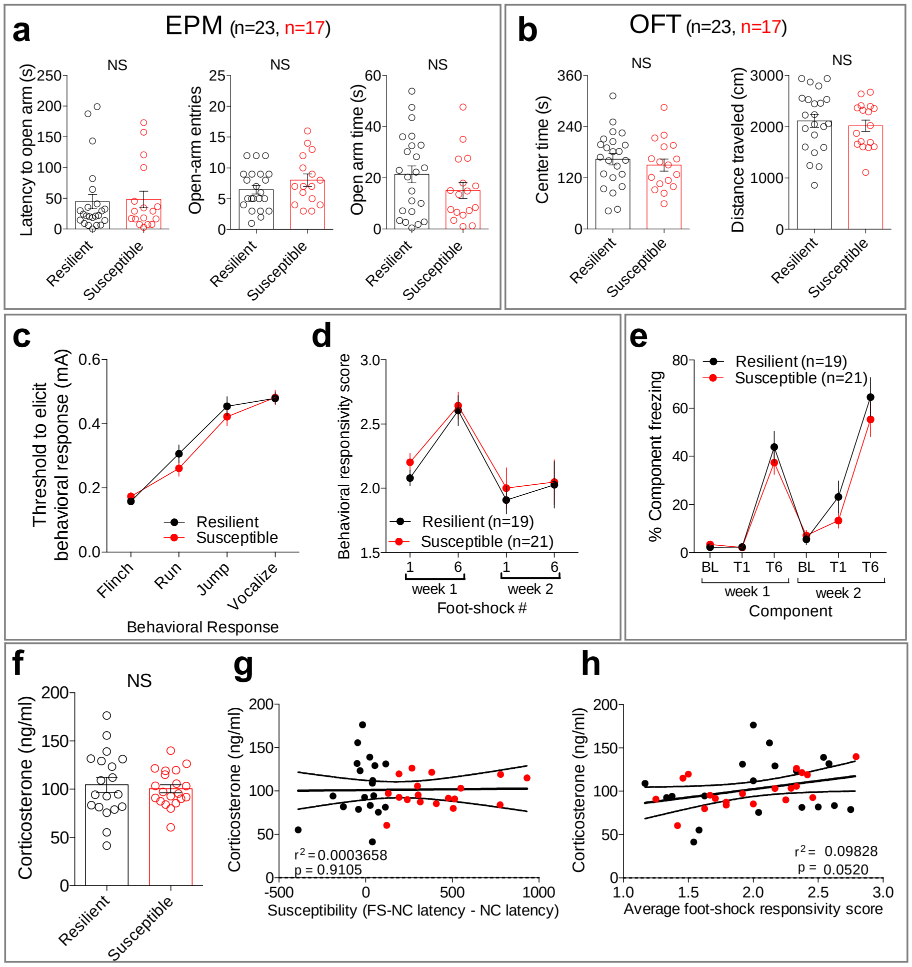

Supplementary Figure 2. Baseline anxiety, foot-shock responsivity, and stress-induced corticosterone release do not differ between susceptible and resilient groups. (a) Resilient and susceptible pre-stress elevated plus maze (EPM) latency to open arm, open arm entries, and open arm time. **(b)** Resilient and susceptible open field test (OFT) center time and distance traveled. **(c)** Resilient and susceptible group average foot-shock current thresholds to elicit specified behavioral responses. Although group sizes were n=19 and n=21 for resilient and susceptible groups, not every individual exhibited every behavioral response. **(d)** Resilient and susceptible average behavioral response scores (1-flinch/walk, 2-run, 3-jump) to the first and 6th/last 2 second 0.7mA shocks for 2 stress exposures 1 week apart. **(e)** Resilient and susceptible percent freezing during baseline (BL), and the first tone (T1) and last tone (T6) of 2 foot-shock stress exposures 1 week apart. **(f)** Resilient and susceptible plasma corticosterone measurements 20 minutes after the initiation of foot-shock stress. **(g)** Correlation between plasma corticosterone levels from panel f and susceptibility as defined by the difference in latency between NIH foot-shock novel cage test (FS-NC) and baseline novel cage test (NC). **(h)** Correlation between plasma corticosterone levels in panel f and average foot-shock responsivity scores across all 6 shocks during week 2 (immediately prior to collection of plasma). Unpaired two-tailed t-test conducted on panels a-b, and f. Two-way ANOVA performed on panels c-e. R^2 and P value for linear regression reported in panels g and h. Data are presented as mean ± SEM.

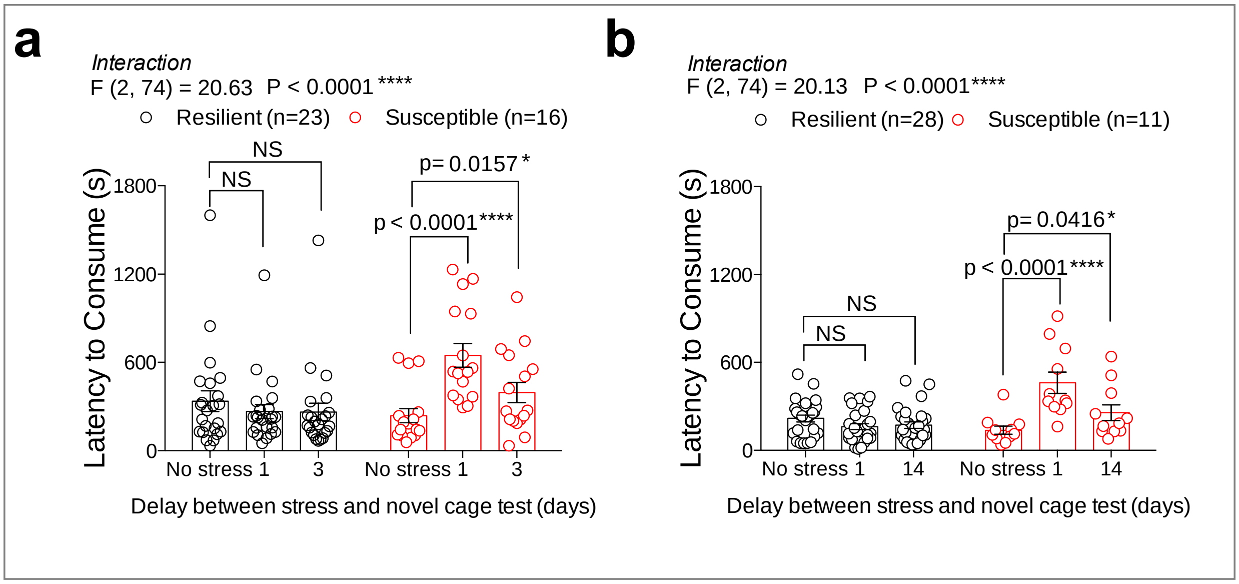

Supplementary Figure 3. Anxiety-like behavior elicited by acute stress is long-lasting. (a) Resilient and susceptible novel cage test latencies in the repeated novelty-induced hypophagia paradigm at baseline (No stress), 1 day after stress, and 3 days or **(b)** 14 days after a second foot-shock stress exposure. F and P values for two-way ANOVA shown above individual panels. P values for pairwise comparisons derived from Holm-Sidak multiple comparisons test after ANOVA shown in panels. Data are presented as mean ± SEM.

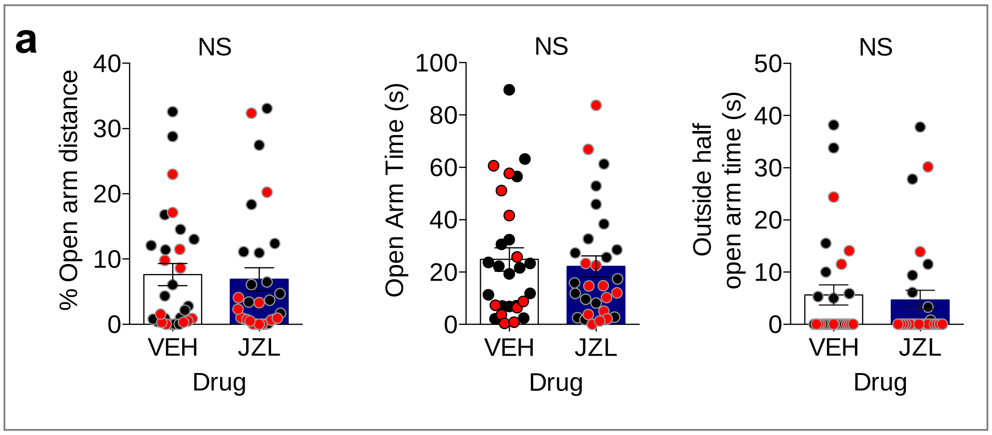

Supplementary Figure 4. JZL-184 does not affect anxiety-like behavior in the elevated plus maze in repeated paradigm. (a) Elevated plus maze % open arm distance (left), open arm time (middle), and time on the outside half of the open arm (right) for resilient (black circles) and susceptible (red circles) individuals 24h after foot-shock stress at least 1 week after susceptibility categorization in the repeated NIH paradigm and treated with either vehicle (VEH) or JZL-184 (JZL; blue bar). Data combined from 2 independent cohorts. Unpaired two-tailed t-tests performed for each measure. Data are presented as mean ± SEM.

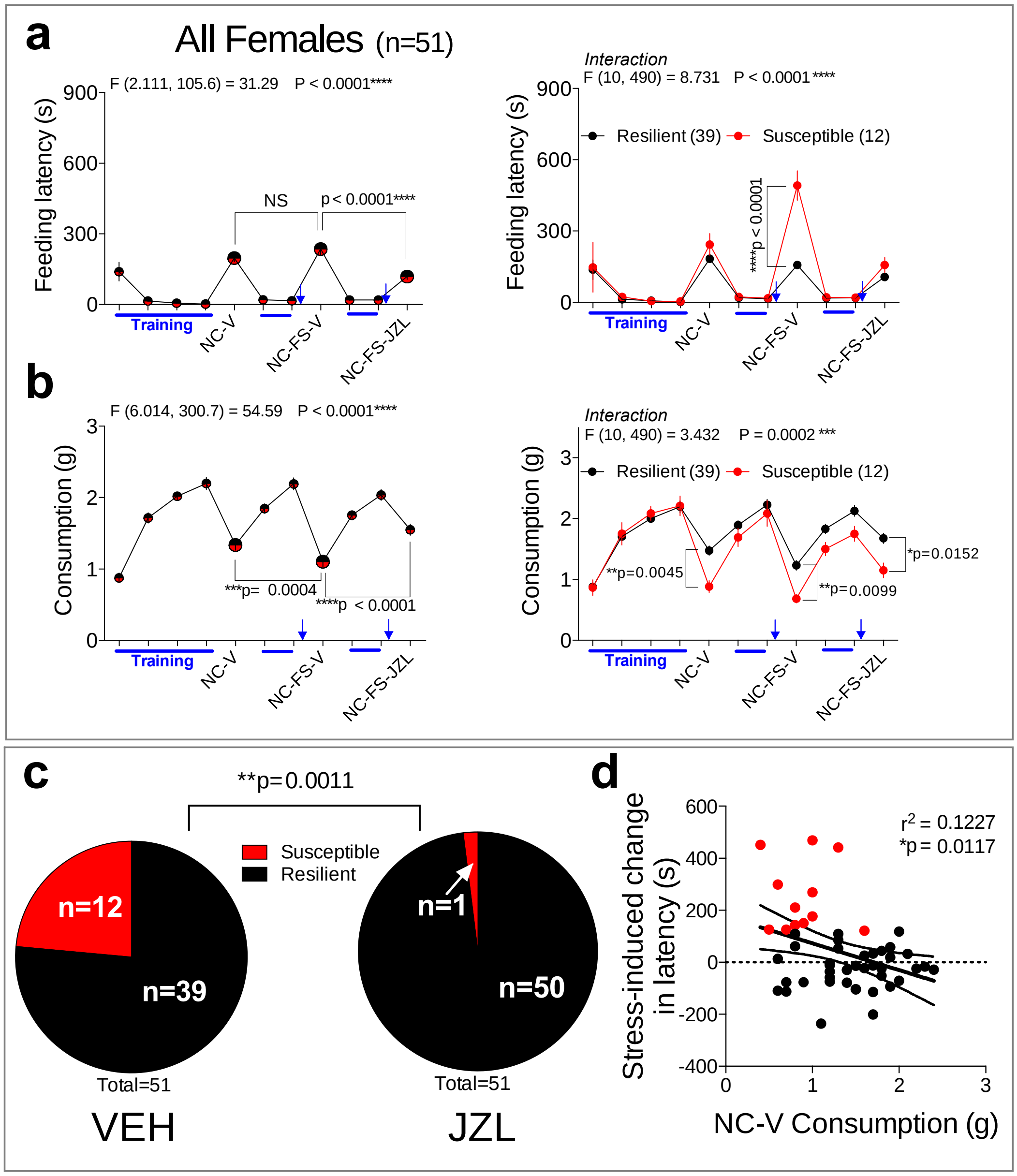

Supplementary Figure 5. JZL-184 promotes resilience in female mice.
(**a**) Female home cage (blue lines) and novel cage (NC) latencies and (**b**) consumption at baseline (NC-V) and after stress with vehicle (NC-FS-V) or JZL (NC-FS-JZL). (**c**) Proportion of susceptible and resilient females after vehicle (VEH) and JZL-184 treatment. (**d**) Correlation between baseline novel cage consumption and stress-induced change in latency from baseline. Blue arrows indicate stress exposure. F and P values for one-way (left) or two-way (right) ANOVA shown above individual panels (a-b). P values for pairwise comparisons derived from Holm-Sidak multiple comparisons test after ANOVA shown in panels. R^2 and P value for linear regression reported in panel (d). P value from chi-squared test reported in panel (c). Data are presented as mean ± SEM.

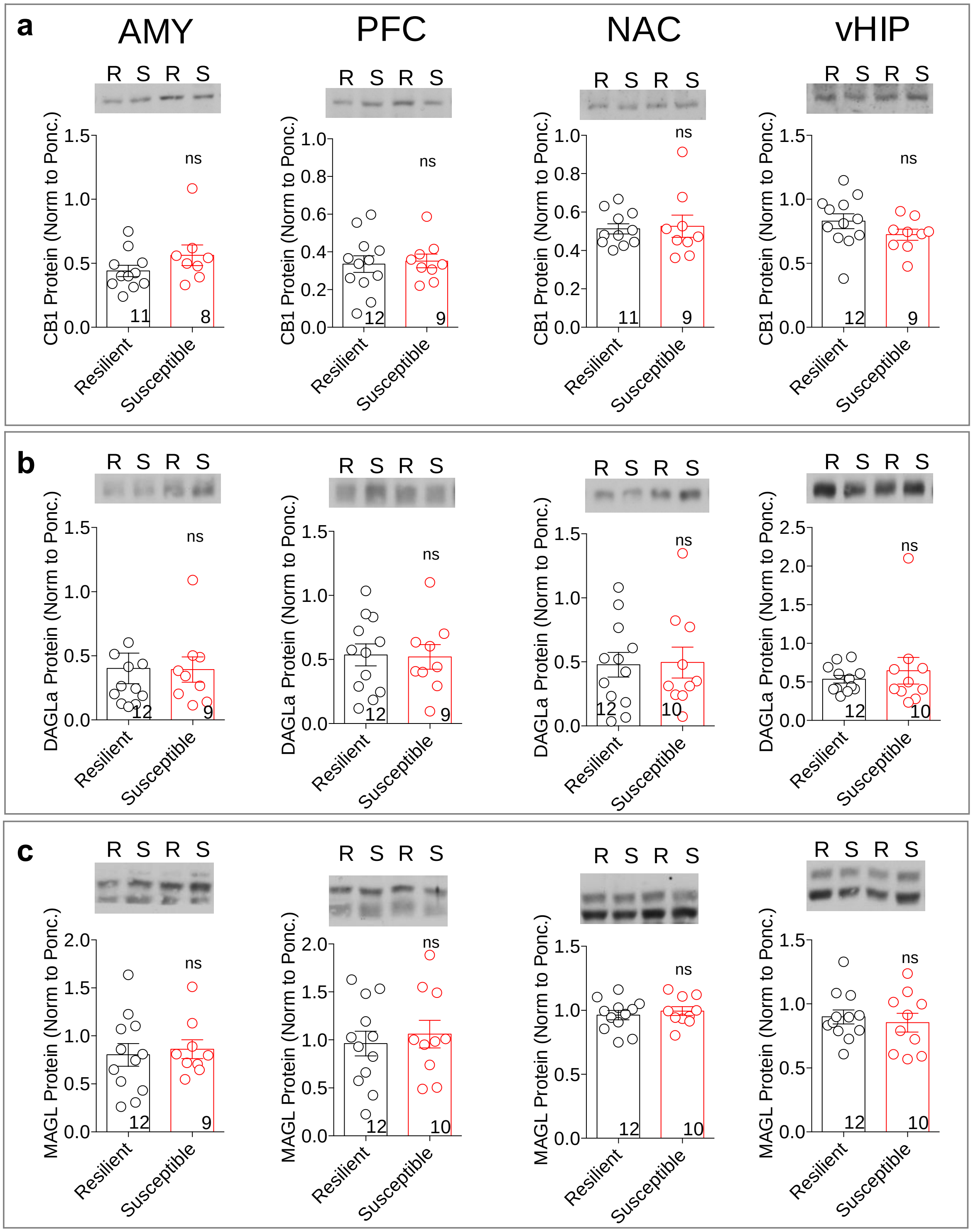

Supplementary Figure 6. CB1 receptor, DAGLα, and MAGL protein levels do not differ between resilient and susceptible groups. (**a**) CB1 receptor protein levels as measured by western blot in the amygdala (AMY), prefrontal cortex (PFC), nucleus accumbens (NAC), and ventral hippocampus (vHIP) for resilient (R) and susceptible (S) mice. (**b**) DAGLα and (**c**) MAGL protein levels in each brain region as measured by western blot. Representative blots shown above each bar graph. Unpaired two-tailed t-tests were performed for each measure. Data are presented as mean ± SEM.

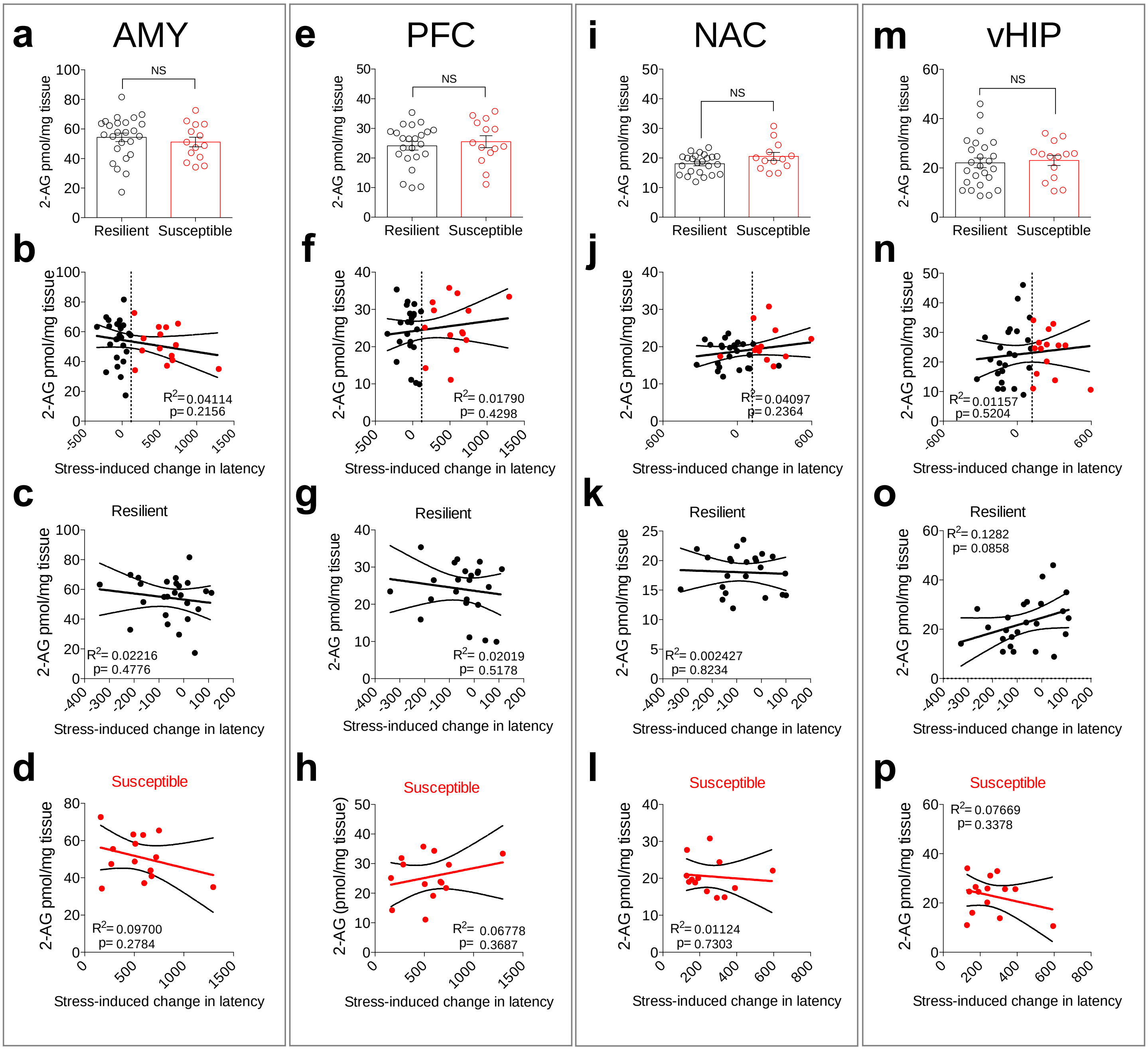

Supplementary Figure 7. 2-AG levels do not correlate with resilience. (**a**) Amygdala (AMY) 2-AG levels for resilient and susceptible groups. (**b**) Correlation between amygdala 2-AG and susceptibility as measured by stress-induced change in latency for the whole population and (**c**) resilient and (**d**) susceptible subpopulations. (**e**) Prefrontal cortex (PFC) 2-AG levels for resilient and susceptible groups. (**f**) Correlation between PFC 2-AG and susceptibility as measured by stress-induced change in latency for the whole population and (**g**) resilient and (**h**) susceptible subpopulations. (**i**) Nucleus accumbens (NAC) 2-AG levels for resilient and susceptible groups. (**j**) Correlation between NAC 2-AG and susceptibility as measured by stress-induced change in latency for the whole population and (**k**) resilient and (**l**) susceptible subpopulations. (**m**) Ventral hippocampus (vHIP) 2-AG levels for resilient and susceptible groups. (**n**) Correlation between vHIP 2-AG and susceptibility as measured by stress-induced change in latency for the whole population and (**o**) resilient and (**p**) susceptible subpopulations. Unpaired two-tailed t-tests were performed for panels a, e, i, and m. R^2 and P value for linear regressions reported in all other panels. Data are presented as mean ± SEM.

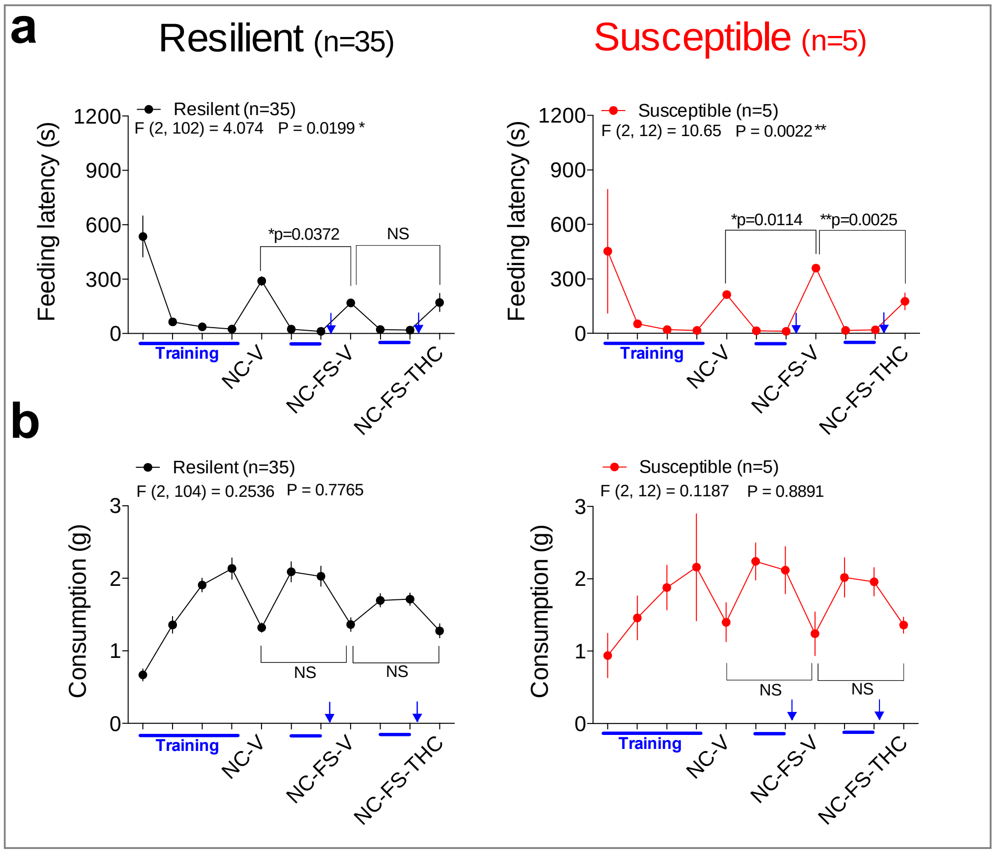

Supplementary Figure 8. THC promotes resilience similarly to JZL-184. (**a**) Male resilient and susceptible home cage (blue lines) and novel cage (NC) latencies and (**b**) consumption at baseline (NC-V) and after stress with vehicle (FS-V) and delta-9-tetrahydrocannabinol (FS-THC). Blue arrows indicate stress exposure. F and P values for one-way ANOVA shown above individual panels. P values for pairwise comparisons derived from Sidak multiple comparisons test after ANOVA shown in panels. Data are presented as mean ± SEM.

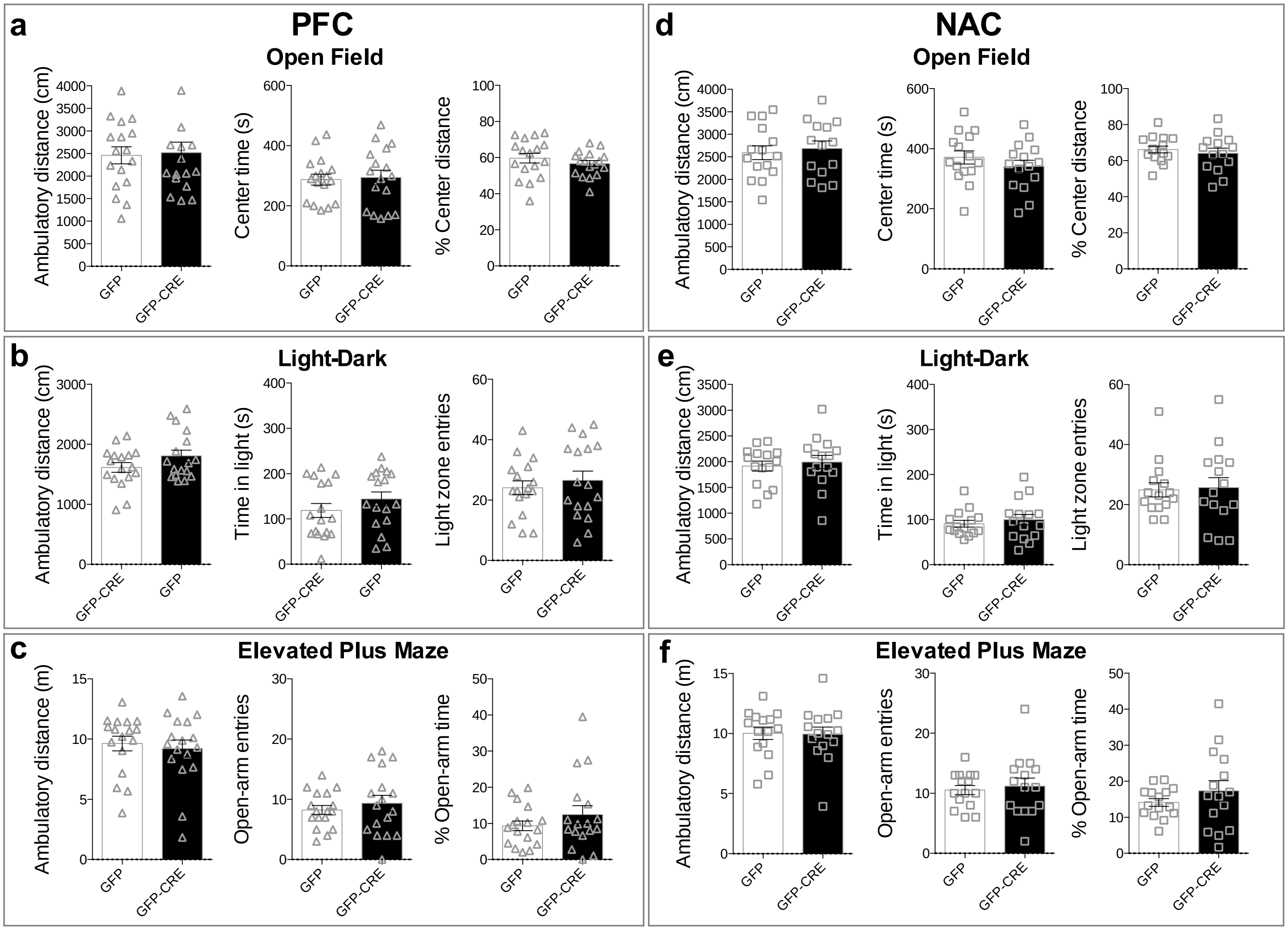

Supplementary Figure 9. Basal anxiety is not significantly affected by loss of PFC or NAc 2-AG. **(a)** Open field test **(b)** light-dark and **(c)** elevated plus maze behavior of prefrontal cortex (PFC) GFP control versus GFP-CRE injected DAGL$^{fl/fl}$ mice. **(d)** Open field test **(e)** light-dark and **(f)** elevated plus maze behavior of nucleus accumbens (NAC) GFP control versus GFP-CRE injected DAGL$^{fl/fl}$ mice. Data for both brain regions was combined from two independent cohorts. Unpaired two-tailed t-tests were performed for each measure; no significant differences were found between groups for any measure. Data are presented as mean ± SEM.

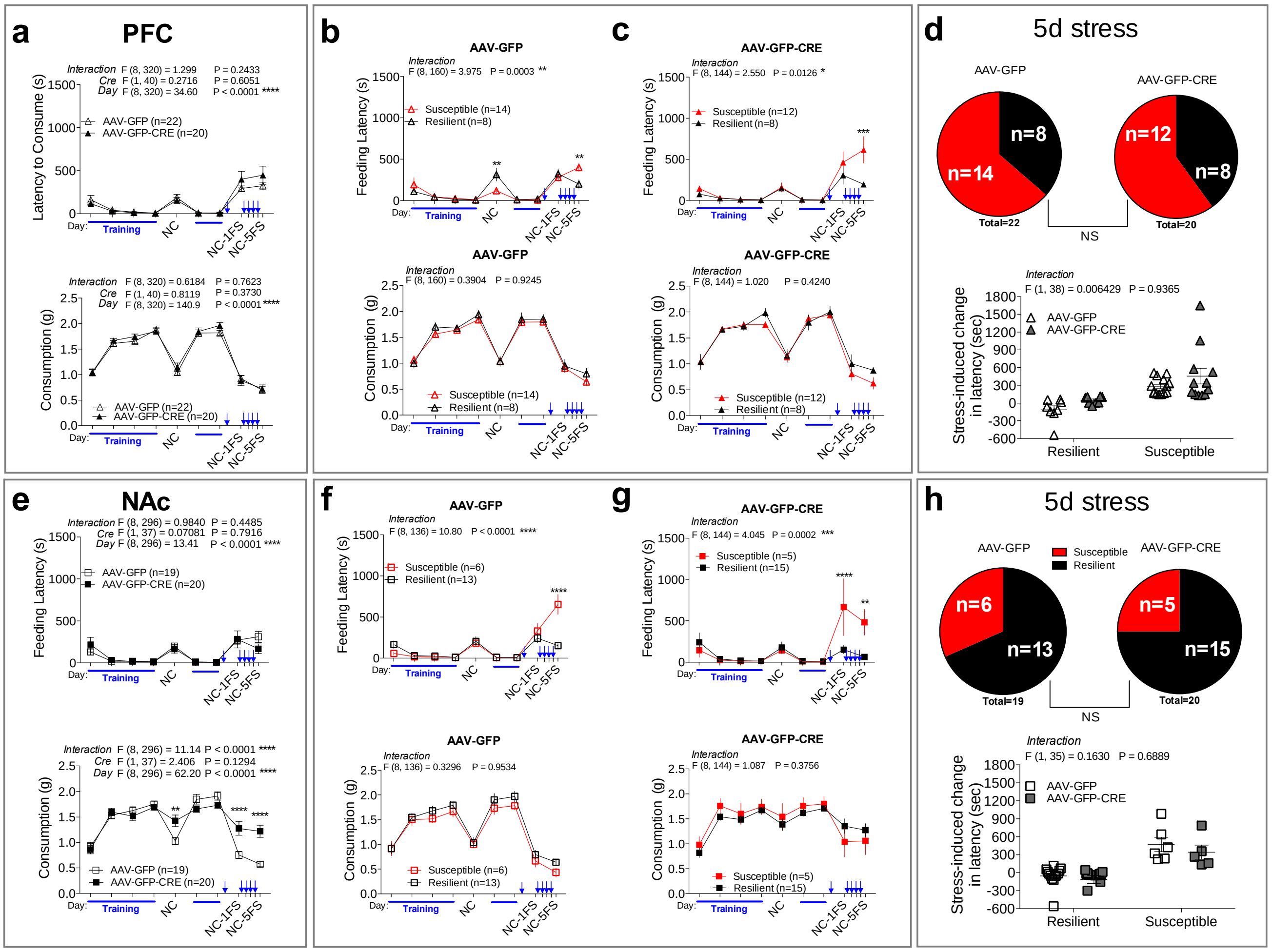

Supplementary Figure 10. Stress susceptibility is not significantly affected by loss of PFC or NAC 2-AG. (**a**) Latency (top) and consumption (bottom) measurements for repeated novelty-induced hypophagia (rNIH) testing of prefrontal cortex (PFC) GFP control versus GFP-CRE injected DAGL$^{fl/fl}$ mice. (**b**) PFC AAV-GFP and (**c**) PFC AAV-GFP-CRE rNIH data from panel (a) split into resilient and susceptible subpopulations using the latency difference between novel cage testing 24h after 5 days of stress exposure (NC-5FS) and baseline (NC). (**d**) Susceptibility ratios for PFC AAV-GFP control versus AAV-GFP-CRE injected mice from panels (b) and (c). (**e**) Latency (top) and consumption (bottom) measurements for repeated novelty-induced hypophagia (rNIH) testing of nucleus accumbens (NAC) GFP control versus GFP-CRE injected DAGL$^{fl/fl}$ mice. (**f**) NAC AAV-GFP and (**g**) NAC AAV-GFP-CRE rNIH data from panel (a) split into resilient and susceptible subpopulations using the latency difference between novel cage testing 24h after 5 days of stress exposure (NC-5FS) and baseline (NC). (**h**) Susceptibility ratios for PFC AAV-GFP control versus AAV-GFP-CRE injected mice from panels (f) and (g). Blue arrows indicate stress exposure which occurred once per day for 5 consecutive days. Data for each brain region was combined from two independent cohorts. F and P values for two-way ANOVA shown in all panels. Asterisks indicate significance as determined by pairwise comparisons from Holm-Sidak multiple comparisons test after ANOVA. Chi-squared tests of susceptibility ratios were not significant (d,h). Data are presented as mean ± SEM.

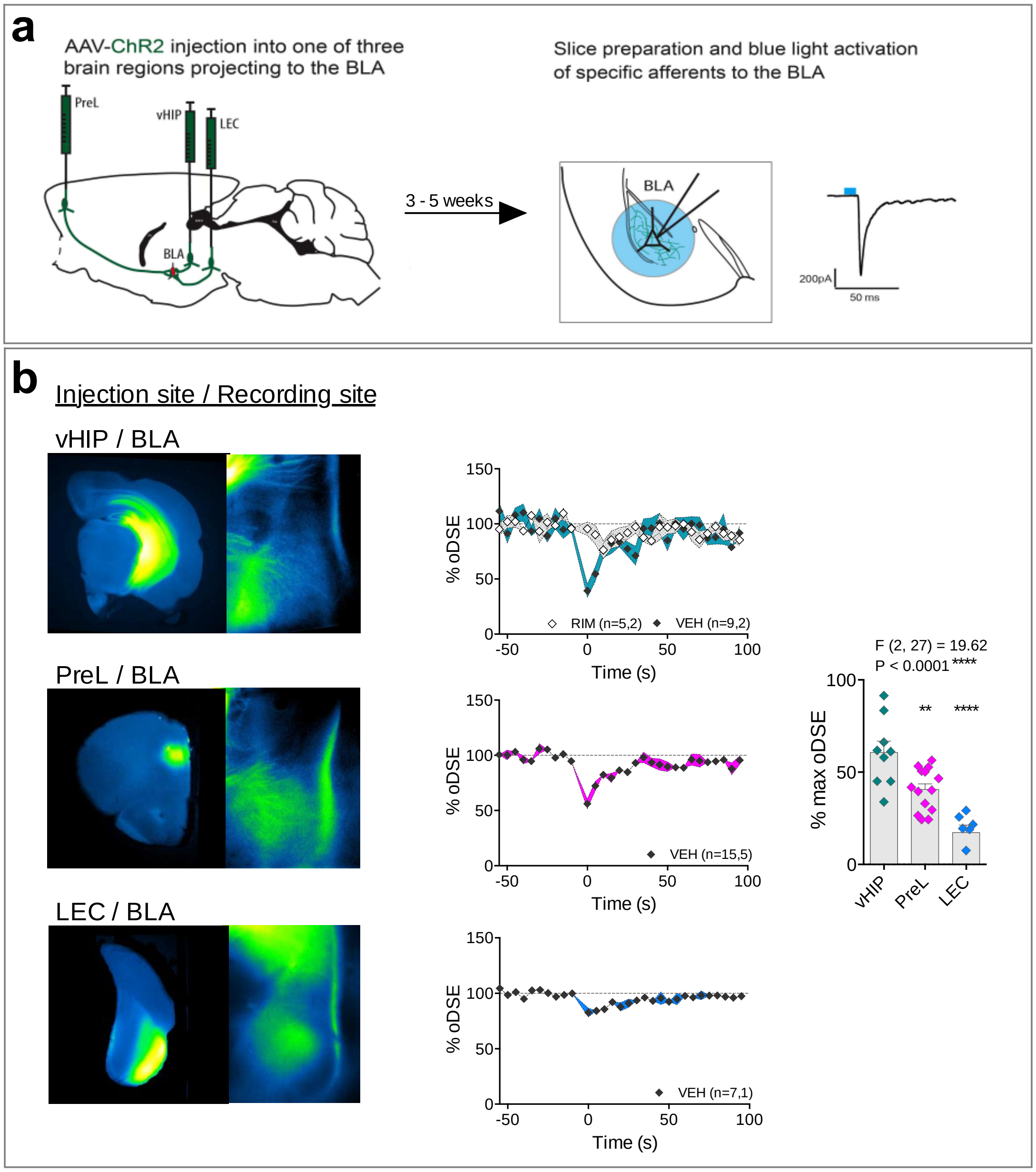

Supplementary Figure 11. 2-AG most strongly modulates ventral hippocampal inputs to the BLA. (a) Schematic figure for afferent-specific electrophysiological recordings in BLA. **(b)** Left: Representative images of YFP labeled injection and recording sites. Middle: Corresponding graphs of depolarization induced suppression of excitation (oDSE) of optically evoked currents from ventral hippocampal (vHIP), prelimbic prefrontal cortical (PreL), and lateral entorhinal cortical (LEC) inputs onto BLA pyramidal cells with vehicle (VEH) incubation. Rimonabant incubation verified that the vHIP-BLA oDSE is CB1-dependent (RIM, grey trace, top middle). Right: comparison of the maximally evoked oDSE of the separate inputs. Number of (cells, animals) are shown for each group. F and P value for one-way ANOVA shown (d). Asterisks indicate significance of P value from Holm-Sidak multiple comparison test between PreL or LEC and vHIP. Data are presented as mean ± SEM.

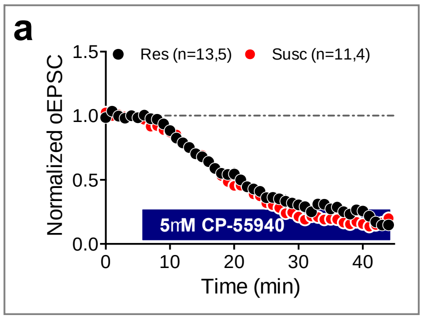

Supplementary Figure 12. CB1 receptor agonist-induced depression in the BLA does not differ between resilient and susceptible mice. (**a**) CP-55940 induced depression of optically evoked EPSCs (oEPSC) from ventral hippocampal synapses in basolateral amygdala (BLA) pyramidal cells. Number of (cells, animals) are shown for each group. Data are presented as mean ± SEM.

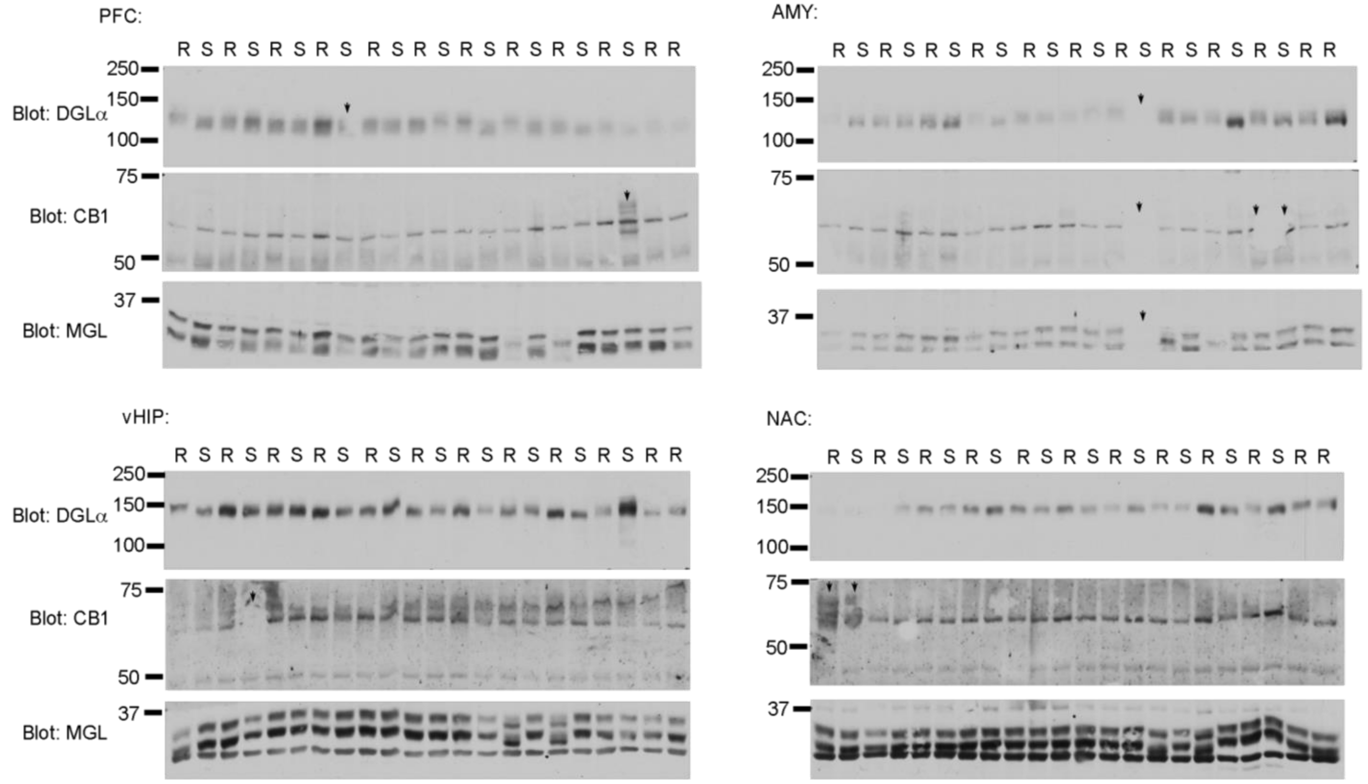

Supplementary Figure 13. Full un-cropped blots are shown corresponding to Supplementary Figure 6. Arrows are shown marking samples that were not included in analysis due to western blot artifact. For MGL density measurements, several bands were present, presumably corresponding to multiple isoforms that were present in the tissue. For consistency, the density of the top two molecular weight isoforms were selected for analysis.
